# mTOR pathway activation in age-related retinal disease

**DOI:** 10.18632/aging.100303

**Published:** 2011-04-03

**Authors:** Chen Zhao, Douglas Vollrath

**Affiliations:** ^1^ Nanjing Medical University, First Affiliated Hospital, Nanjing, 210029, China; ^2^ Tianjin Key Laboratory of Ophthalmology and Vision Science, Tianjin Eye Hospital, Tianjin, 300020, China; ^3^ Department of Genetics, Stanford University School of Medicine, Stanford, CA 94305, USA

Vision loss degrades the quality of life of aged individuals. The major cause in industrialized countries is age-related macular degeneration (AMD), a blinding eye disease due to death of photoreceptors in the macula, a specialized retinal region responsible for high acuity vision. Photoreceptor death in AMD is thought to follow damage to the retinal pigment epithelium (RPE) [1], a monolayer of polarized, post-mitotic cells located between the photoreceptors and the choroidal blood supply that performs a variety of crucial tasks [2]. One proposed mechanism of RPE dysfunction in AMD posits a lifetime of oxidative damage leading to deposits (termed drusen) between the RPE and choroid, inflammation [3], and diminished RPE mitochondrial function [4, 5]. Macular RPE mitochondrial DNA from AMD eyes is more damaged than corresponding macular nuclear DNA [6], and macular RPE mitochondrial DNA damage correlates positively with AMD severity [7]. To model RPE mitochondrial DNA damage in AMD, we selectively ablated mitochondrial DNA replication and transcription in the RPE of postnatal mice [8]. The resulting deficit in RPE oxidative phosphorylation (OXPHOS) caused a slowly progressive photoreceptor degeneration, as well as a number of RPE morphological changes similar to those seen in AMD. The most prominent early RPE changes were hypertrophy and dedifferentiation, which coincided with activation of the mTOR pathway in OXPHOS-deficient RPE cells.

Robust mTOR activation in the context of OXPHOS deficiency is counterintuitive because mTOR integrates trophic factor and nutrient availability signals to regulate cell growth and proliferation [9], and poisoning of mitochondrial energy production inhibits mTOR [10]. The fact that ATP levels in OXPHOS-deficient RPE cells were not substantially different from controls helps to resolve this apparent paradox. Levels of selected glycolytic metabolites were increased by several orders of magnitude, indicating a large glycolytic flux capable of generating ATP at a high rate. However, dependence on aerobic glycolysis is not a requirement for mTOR activation; acute treatment of wild-type mice with a strong oxidant that the targets the RPE also activated mTOR and triggered dedifferentiation, with profound negative consequences for adjacent photoreceptors [8]. Features suggestive of RPE hypertrophy and/or dedifferentiation have been reported for a number of other mouse retinal degeneration models [11-13], suggesting that a mTOR-associated RPE stress response may be quite general.

OXPHOS deficiency leads eventually to RPE atrophy, which is seen more commonly in AMD than RPE hypertrophy. Our findings suggest that RPE hypertrophy may be present at earlier stages of AMD. Indeed, ocular coherence tomography imaging demonstrated thickened macular RPE more frequently in early AMD eyes than in advanced AMD or control eyes (C. Zhao, unpublished). RPE hypertrophy may be less prominent in advanced AMD because drusen and diminished transport through aged Bruch's basement membrane [14] may restrict access of RPE cells to nutrients from the choroidal blood supply. RPE cells in most mouse models are presumably not limited in this regard, facilitating mTOR activation. Hence, the stress response we have identified may shed light on RPE-related disease processes in which nutrients are readily available.

Intriguingly, pharmacological inhibition of mTORC1 with rapamycin blunted RPE dedifferentiation and hypertrophy and preserved photoreceptor numbers and function for both the metabolic and oxidative stress models [8]. Rapamycin has recently been shown to have the remarkable ability to increase the longevity of mice, even when administered late in life [15]. Our results thus connect age-dependent retinal degeneration with a pathway known to be critical for the determination of lifespan. An in depth understanding is needed of the requirements for mTOR activation in the RPE and the mechanism by which the pathway mediates RPE dedifferentiation, with the goal of combating age-related retinal disease and extending human healthspan.

## References

[R1] Zarbin MA (2004). Current concepts in the pathogenesis of age-related macular degeneration. Arch Ophthalmol.

[R2] Marmor MF, Wolfensberger WT (1999). The Retinal Pigment Epithelium Function and Disease.

[R3] Hageman GS, Luthert PJ, Victor Chong NH, Johnson LV, Anderson DH, Mullins RF (2001). An integrated hypothesis that considers drusen as biomarkers of immune-mediated processes at the RPE-Bruch's membrane interface in aging and age-related macular degeneration. Prog Retin Eye Res.

[R4] Cai J, Nelson KC, Wu M, Sternberg P, Jones DP (2000). Oxidative damage and protection of the RPE. Prog Retin Eye Res.

[R5] Jarrett SG, Lin H, Godley BF, Boulton ME (2008). Mitochondrial DNA damage and its potential role in retinal degeneration. Prog Retin Eye Res.

[R6] Karunadharma PP, Nordgaard CL, Olsen TW, Ferrington DA (2011). Mitochondrial DNA damage as a potential mechanism for age-related macular degeneration. Invest Ophthalmol Vis Sci.

[R7] Lin H, Xu H, Liang FQ, Liang H, Gupta P, Havey AN, Boulton ME, Godley BF (2011). Mitochondrial DNA damage and repair in RPE associated with aging and age-related macular degeneration. Invest Ophthalmol Vis Sci.

[R8] Zhao C, Yasumura D, Li X, Matthes M, Lloyd M, Nielsen G, Ahern K, Snyder M, Bok D, Dunaief JL (2011). mTOR-mediated dedifferentiation of the retinal pigment epithelium initiates photoreceptor degeneration in mice. J Clin Invest.

[R9] Zoncu R, Efeyan A, Sabatini DM (2011). mTOR: from growth signal integration to cancer, diabetes and ageing. Nat Rev Mol Cell Biol.

[R10] Dennis PB, Jaeschke A, Saitoh M, Fowler B, Kozma SC, Thomas G (2001). Mammalian TOR: a homeostatic ATP sensor. Science.

[R11] Justilien V, Pang JJ, Renganathan K, Zhan X, Crabb JW, Kim SR, Sparrow JR, Hauswirth WW, Lewin AS (2007). SOD2 knockdown mouse model of early AMD. Invest Ophthalmol Vis Sci.

[R12] Rattner A, Toulabi L, Williams J, Yu H, Nathans J (2008). The genomic response of the retinal pigment epithelium to light damage and retinal detachment. J Neurosci.

[R13] Bruban J, Glotin AL, Dinet V, Chalour N, Sennlaub F, Jonet L, An N, Faussat AM, Mascarelli F (2009). Amyloid-beta(1-42) alters structure and function of retinal pigmented epithelial cells. Aging Cell.

[R14] Hussain AA, Rowe L, Marshall J (2002). Age-related alterations in the diffusional transport of amino acids across the human Bruch's-choroid complex. J Opt Soc Am A Opt Image Sci Vis.

[R15] Harrison DE, Strong R, Sharp ZD, Nelson JF, Astle CM, Flurkey K, Nadon NL, Wilkinson JE, Frenkel K, Carter CS (2009). Rapamycin fed late in life extends lifespan in genetically heterogeneous mice. Nature.

